# Persisting Microbiota and Neuronal Imbalance Following *T. gondii* Infection Reliant on the Infection Route

**DOI:** 10.3389/fimmu.2022.920658

**Published:** 2022-07-11

**Authors:** Timothy French, Johannes Steffen, Albert Glas, Lisa Osbelt, Till Strowig, Björn H. Schott, Thomas Schüler, Ildiko Rita Dunay

**Affiliations:** ^1^ Institute of Inflammation and Neurodegeneration, Health Campus Immunology, Infectiology and Inflammation (GC-I), Otto-von-Guericke University, Magdeburg, Germany; ^2^ Department of Microbial Immune Regulation, Helmholtz Centre for Infection Research, Braunschweig, Germany; ^3^ Center for Behavioral Brain Sciences, Magdeburg, Germany; ^4^ Department of Psychiatry and Psychotherapy, University Medical Center Göttingen, Göttingen, Germany; ^5^ Leibniz Institute for Neurobiology, Magdeburg, Germany; ^6^ Institute of Molecular and Clinical Immunology, Otto-von-Guericke-University Magdeburg, Magdeburg, Germany

**Keywords:** *Toxoplasma gondii*, cerebral toxoplasmosis, neuroinflammation, microbiota, infection route

## Abstract

*Toxoplasma gondii* is a highly successful parasite capable of infecting all warm-blooded animals. The natural way of infection in intermediate hosts is the oral ingestion of parasite-contaminated water or food. In murine experimental models, oral infection (*p.o.*) of mice with *T. gondii* is applied to investigate mucosal and peripheral immune cell dynamics, whereas *intraperitoneal* infection (*i.p.*) is frequently used to study peripheral inflammation as well as immune cell – neuronal interaction in the central nervous system (CNS). However, the two infection routes have not yet been systematically compared along the course of infection. Here, C57BL/6 mice were infected *p.o.* or *i.p.* with a low dose of *T. gondii* cysts, and the acute and chronic stages of infection were compared. A more severe course of infection was detected following *i.p.* challenge, characterized by an increased weight loss and marked expression of proinflammatory cytokines particularly in the CNS during the chronic stage. The elevated proinflammatory cytokine expression in the ileum was more prominent after *p.o.* challenge that continued following the acute phase in both *i.p.* or *p.o.* infected mice. This resulted in sustained microbial dysbiosis, especially after *p.o.* challenge, highlighted by increased abundance of pathobionts from the phyla proteobacteria and a reduction of beneficial commensal species. Further, we revealed that in the CNS of *i.p.* infected mice CD4 and CD8 T cells displayed higher IFNγ production in the chronic stage. This corresponded with an increased expression of C1q and CD68 in the CNS and reduced expression of genes involved in neuronal signal transmission. Neuroinflammation-associated synaptic alterations, especially PSD-95, VGLUT, and EAAT2 expression, were more pronounced in the cortex upon *i.p.* infection highlighting the profound interplay between peripheral inflammation and CNS homeostasis.

## Introduction

Over a myriad of years, the apicomplexan parasite, *Toxoplasma gondii*, has evolved strategies to survive oral digestion and pervade the biological barriers of the body. This highly successful parasite infects roughly 30%-70% of the human population, depending on age and geographic location (1, 2). This has led to *T. gondii* becoming a well-accepted model of infection, to study different topics ranging from the type 1 (Th1) intracellular immune response, host-pathogen interaction, and irritable bowel syndrome (IBS) to chronic infection-induced neuroinflammation (3–11). These laboratory studies use either intraperitoneal (*i.p.*) or peroral (*p.o.*) injections, depending on the anatomical location that they primarily aim to investigate. Intraperitoneal infections bypass many of the initial obstacles inherent to the natural oral route. Studies that focus on mucosal immune responses or the influence of the microbiome typically use the oral injection route. Information directed at the difference between long-term effects of peritoneal versus oral invasion is rather limited.

Chronic *T. gondii* infection is associated with subtle alterations in the host´s immune and nervous system. Previous studies suggested that the persisting neuroinflammation might be responsible for these distinct changes in the host’s behavior (12–16). Seropositive individuals have been shown to exhibit subclinical symptoms as well as an increased risk for neuropsychiatric disorders such as epilepsy, seizures, or schizophrenia (17–24). Conclusively, a recent large-scale study provided compelling evidence for the association between latent *T. gondii* infection and schizophrenia in humans (1). Distinctly, the immune response to *T. gondii* and the neuropathological mechanisms of these disorders overlap by involving the same cell types and, at least in part, the same molecules (24–27). We have previously demonstrated that chronic *T. gondii* infection leads to a loss of structural complexity of axons and dendrites, primarily in the neocortex and hippocampus (14). Moreover, we have recently shown that the synaptic protein composition was altered, in particular, with a downregulation of components of glutamatergic signaling as a consequence of the inflammatory milieu induced by chronic infection (28, 29).

Previous studies have described differences between the infection types in the early detection and induction of adaptive immunity depending on the infection route (30, 31). Here we aimed to investigate the influence of the infection route on the gut microbiota, neuroinflammation, and subsequent neuronal signaling in the acute and chronic phases of the infection. We demonstrate that *i.p.* infected mice developed long-term dysbiosis and a more severe course of proinflammatory response as well as enhanced reduction of gene expression that are essentially involved in neural signal transmission.

## Methods

### Animals

Experiments were conducted using female, wild type (WT) C57BL/6J mice (8 weeks old; purchased from Janvier Laboratories, Cedex, France). Mice were kept under specific pathogen-free (SPF) conditions. Four to five mice were used for all experimental groups. Non-infected, non-treated mice were used as the control group. All data are representative of three independent experiments. All mice were group-housed in 12h day/night cycles at 22 °C with free access to food and water. Experiments were approved by local authorities according to German and European legislation.

### *Toxoplasma gondii* Infection

*T. gondii* cysts of type II strain ME49 were harvested from the brains of female NMRI mice infected with *T. gondii* cysts 6-10 months earlier, as described previously (28). In short, isolated brains were mechanically homogenized in 1 ml sterile phosphate-buffered saline (PBS), and the number of cysts in the homogenate was determined using a light microscope. Mice were infected with two cysts intraperitoneal or *via* oral gavage.

### Organ Collection

First, mice were deeply anesthetized by isoflurane inhalation (Baxter, Halle/Westfallen, Germany). Subsequently, mice were transcardially perfused with sterile ice-cold PBS. Single-cell suspension of mesenteric lymph nodes and spleen were generated by mechanically passing tissue through a 40 μm strainer in PBS complemented with 2% fetal calf serum (FCS). Samples stored in RNA*later* (Qiagen, Stockach, Germany) were kept at 4°C overnight and then transferred to -20°C.

### Cell Isolation

To isolate brain immune cells, brains were homogenized in a buffer containing 1 M HEPES (pH 7.3) and 45% glucose and then filtered through a 70 µm strainer. Leukocytes were separated *via* Percoll density gradient centrifugation (GE Healthcare, Pasching, Austria) as described previously (32). Living cells were counted using a Neubauer chamber and trypan blue staining.

### Flow Cytometric Analysis

Single-cell suspensions were incubated with an anti-FcγIII/II receptor antibody (clone 93, eBioscience, Frankfurt, Germany) to block unspecific binding and Zombie NIR™ (BioLegend, Koblenz, Germany), a fixable viability dye. Thereafter, cells were stained with fluorochrome-conjugated antibodies against cell surface markers: CD45 (30-F11), CD11b (M1/70), Ly6C (HK1.4), MHCI (28-14-8), and MHCII (M5/114.15.2), all purchased from eBioscience; CD3 (17A2), CD4 (RM4-5), CD8α (53-6.7), and CD80 (16-10A1) all purchased from Biolegend; and Ly6G (1A8) purchased from BD Biosciences in FACS buffer (with 2% FBS, 0.1% NaN3) at 4°C for 30 min and then fixed in 4% paraformaldehyde (PFA, Affymetrix, Santa Clara, CA, USA) for 15 min. Matched FMO controls were used to assess the level of background fluorescence in the respective detection channel.

Expression of cytokines was evaluated by *ex vivo* restimulation with *Toxoplasma* lysate antigen (TLA). In short, 5x105 cells were resuspended in DMEM, supplemented with 10% FCS and 1% Pen/Strep, and stimulated with 200 µg/mL TLA for 6h at 37°C, 5% CO2. After 2h of stimulation, a combination of brefeldin A (10 µg/mL, BioLegend, Koblenz, Germany) and monensin (10 µg/mL, BioLegend, Koblenz, Germany) was added. Cells were stained for cell surface markers as described above. Stained cells were then fixed in 4% PFA for 15 min and washed twice with Permeabilization buffer eBioscience, Frankfurt, Germany). Cells were then stained against IFNγ for 45 min at 4°C, and resuspended in FACS buffer for acquisition. Matched isotype controls were used to assess the level of non-specific binding.

Flow cytometric analysis was performed on Attune NxT Flow Cytometer (Thermo Fisher) and analyzed with FlowJo (version 10, Flowjo LLC).

### DNA and RNA Isolation

Samples stored in RNA*later^®^* were homogenized in BashingBeads tubes (Qiagen, Stockach, Germany). AllPrep DNA/RNA Mini Kit (Qiagen, Stockach, Germany) was used to isolate DNA and the peqGOLD total RNA kit (Peqlab, Erlangen, Germany) was used to isolate total RNA from the homogenate following the manufacturer’s instructions.

### Semiquantitative RT-qPCR

*T. gondii* burden was determined using the FastStart Essential DNA Green Master kit (Roche, Penzberg, Germany). *T. gondii* B1 gene was used as target (Tg. B1), and *Mus musculus Asl* gene (Mm. Asl) was used as a reference. Parasite stage was quantified using bradyzoite-specific *Bag1* and tachyzoite-specific *Sag1* as targets and *Gapdh* as reference gene (Power SYBR^®^ Green RNA-to-CT™ 1-Step Kit, Thermo Fisher, Dreieich, Germany) for. All primers were purchased from TIBMolbiol, Berlin, Germany.

Relative gene expression was determined similar to previous descriptions (33, 34) using the TaqMan^®^ RNA-to-CT™ 1-Step Kit (life technologies, Darmstadt, Germany). TaqMan^®^ Gene Expression Assays (life technologies) were used for mRNA amplification of *Ifng* (Mm00801778_m1), *Tnf* (Mm00443258_m1), *Il12a* (Mm00434165_m1), *Nos2* (Mm00440485_m1). Expression of *Hprt* (Mm01545399_m1) was chosen as reference and target/reference ratios were calculated with the LightCycler^®^ 96 software version 1.1 (Roche, Penzberg, Germany). All results were further normalized to the mean of the wild type infected group.

### Cytokine and Chemokine Assessment

Cytokine and chemokine profile was characterized using the LEGENDplex™ system (BioLegend, Koblenz, Germany). A more detailed protocol is published (35). Briefly, we used the Mouse Inflammation Panel (13-plex). Serum isolated from WT infected mice was incubated with fluorescence-encoded capture beads to cytokine and chemokine targets, including TNF, IL-1β, IL-6, and IFNγ. The fluorescent signal of analyte-specific bead regions was quantified using flow cytometry, and the concentrations of particular analytes were determined using provided data analysis software (BioLegend, LegendPlex™ software v8.0).

### DNA Isolation From Intestinal Content for Microbiota Analysis

Content samples were collected from the terminal Ileum and stored at -20°C until DNA extraction. DNA was extracted using a phenol-chloroform-based method previously described (36). In brief, 500 µL of extraction buffer (200 mM Tris Roth, Dautphetal, Germany), pH 8.0), 200 µL of 20% SDS Applichem, Darmstadt, Germany), 500 µL of phenol:chloroform:isoamyl alcohol (PCI) (24:24:1) Roth, Dautphetal, Germany) were added per sample. Lysis of bacteria was performed by mechanical disruption using a Mini-BeadBeater-96 (BioSpec, Berlin, Germany) for two times 2 min. After centrifugation, aqueous phase was passed for another phenol:chloroform:isoamyl alcohol extraction before precipitation of DNA using 500 µL isopropanol (J. T. Baker, Radnor, Pennsylvania, USA) and 0.1 volume of 3 M sodium acetate (Applichem, Darmstadt, Germany). Samples were incubated at -20°C for at least several hours or overnight and centrifuged at 4°C at maximum speed for 20 min. Resulting DNA pellet was washed, dried using a speed vacuum, and resuspended in TE Buffer (Applichem) with 100 µg/ml RNase I (Applichem, Darmstadt, Germany). Crude DNA was column purified (BioBasic Inc., Braunschweig, Germany) to remove PCR inhibitors.

### 16S rRNA Gene Amplification and Sequencing

16S rRNA gene amplification of the V4 region (F515/R806) was performed according to an established protocol previously described (37). Briefly, DNA was normalized to 25 ng/µl and used for sequencing PCR with unique 12-base Golary barcodes incorporated *via* specific primers (obtained from Sigma, Steinheim am Albuch, Germany). PCR was performed using Q5 polymerase (New England Biolabs, Frankfurt/Main, Germany) in triplicates for each sample, using PCR conditions of initial denaturation for 30 s at 98°C, followed by 25 cycles (10 s at 98°C, 20 s at 55°C, and 20 s at 72°C). After pooling and normalization to 10 nM, PCR amplicons were sequenced on an Illumina MiSeq platform *via* 250 bp paired-end sequencing (PE250). Using Usearch8.1 software package (http://www.drive5.com/usearch/) the resulting reads were assembled, filtered, and clustered. Sequences were filtered for low quality reads and binned based on sample-specific barcodes using QIIME v1.8.0 (38). Merging was performed using -fastq_mergepairs – with fastq_maxdiffs 30. Quality filtering was conducted with fastq_filter (-fastq_maxee 1), using a minimum read length of 250 bp and a minimum number of reads per sample = 1000. Reads were clustered into 97% ID OTUs by open-reference OTU picking and representative sequences were determined by use of UPARSE algorithm (39). Abundance filtering (OTUs cluster >0.5%) and taxonomic classification were performed using the RDP Classifier executed at 80% bootstrap confidence cut off (40). Sequences without matching reference dataset were assembled as *de novo* using UCLUST. Phylogenetic relationships between OTUs were determined using FastTree to the PyNAST alignment (41). Resulting OTU absolute abundance table and mapping file were used for statistical analyses and data visualization in the R statistical programming environment package phyloseq (42).

### Statistical Analysis

With the exception of microbiome data, all datasets were statistically analyzed using GraphPad Prism 7.02 (GraphPad software). We used either two-way or one-way ANOVA with uncorrected Fischer’s LSD tests or unpaired two-tailed Student’s *t*-tests. Owing to the small sample sizes, unequal variances were assumed in all *t*-tests. The significance level was set to *p < 0.05, **p<0.01, ***p<0.001 for all statistical comparisons, and two-tailed *p*-values are reported for all *t*-tests. Data are presented as mean **±** SEM.

Statistical analyses of microbiome data were performed using R v3.6.1 and the packages phyloseq (42) and ggplot2 (43). For Mann-Whitney U tests, p values lower than 0.05 were considered as significant after multiple testing correction (Benjamini-Hochberg false discovery rate correction). The permutational multivariate ANOVA (ADONIS) were computed with 999 permutations. For ADONIS tests, an R2 > 0.1 (effect size, 10%) and p value < 0.05 were considered as significant.

## Results

### *I.p. T. gondii* Infected Mice Display a More Severe Course of Infection *via* Increased Weight Loss and Parasite Spread

**Figure 1:**
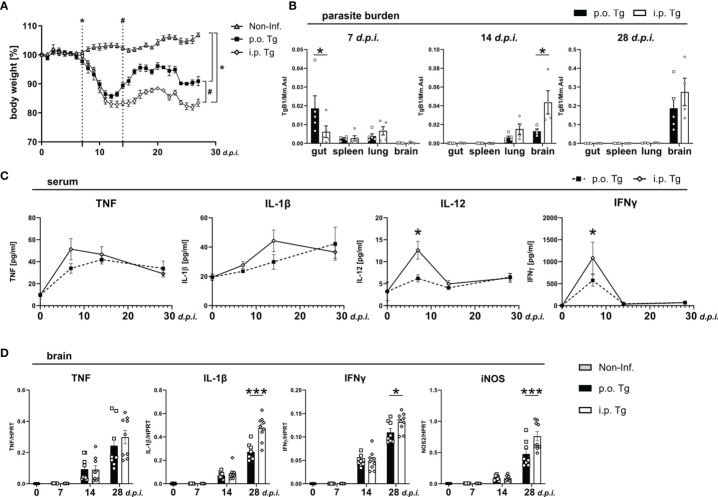
Increased weight loss, parasite spread, and immune response in *i.p. T. gondii* infected mice. Wild-type mice were infected orally (*p.o.* Tg) or *intraperitoneally* (*i.p.* Tg) with *T. gondii* (two cyst, ME49) and **(A)** weighed daily. Differences between non-infected (Non-Inf.) and *T. gondii* infected animals (indicated by *, significant from 7 *days post-infection* as well as *p.o.* and *i.p.* infected animals (indicated by #; significant from day 14 *p.i.*) were analyzed. **(B)** Parasite burden was analyzed in the ileum (gut), spleen, lung, and brain of *p.o.* and *i.p.* infected animals. **(C)** Levels if indicated cytokines were determined in sera f naïve mice (d0) and *T. gondii* infected animals (7, 14, and 28 d*.p.i.*) mice. **(D)** mRNA levels of indicated genes were determined in the brain. Data are presented as mean ± SEM. Differences between groups were analyzed by two-way ANOVA followed by Tukey’s multiple comparisons test (*p<0.05, ***p<0.001).

The environments *T. gondii* encounters when entering the digestive tract versus the peritoneal cavity differ fundamentally. In order to determine if this difference in the barrier to entry affects the course of infection, we infected mice with a low-dose, two cyst (ME49) infection either orally (*p.o.* Tg) or intraperitoneally (*i.p.* Tg). All mice were weighed daily until 28 *days post infection* (*d.p.i.*) (**Figure 1A**). Compared to naïve animals, *p.o.* infected mice began to lose weight at 7 d*.p.i.* (dotted line with *), which remained significantly lower until the end of the experiment (28 d*.p.i.*). Mice infected *i.p.* began to lose weight a day later at 8 d*.p.i.*, which also remained significantly lower until the end of the experiment. When comparing the weight change between the infection routes, we observed a higher gain of weight by *p.o.* infected animals starting at 14 d*.p.i.* that persisted until the end of the experiment (dotted line with #). The difference in weight recovery suggests an enhanced inflammatory response upon *i.p.* infection, which persists over time. In order to determine if the route of infection is affecting the parasites viability and overall associated immune response, we measured the parasite burden in the terminal ileum, spleen, lung, and brain of mice at 7, 14, and 28 d*.p.i.* These dates were chosen for two primary reasons. First, they were critical days in the weight change. Second, these time points coincide with the early, acute, and chronic phases of infection. As expected, we observed a significantly higher parasite burden in the ileum of *p.o.* infected mice, while the burden in spleen, lung, and brain were comparable (**Figure 1B**). Parasites were also detected in the ileum after *i.p.* challenge. By 14 d*.p.i.*, the parasite was primarily found in lung and brain tissue as it penetrated the deeper tissues. In *i.p.* infected mice, there was a higher parasite burden in the lung and a significantly higher burden in the brain (**Figure 1B**). At 28 d*.p.i.*, parasites were not detectable in the peripheral organs and were mainly found in the brain (**Figure 1B**). Similar to 14 d*.p.i.*, there was a trend of higher parasite burden in the brains of *i.p.* infected mice.

Next, we measured the blood cytokine levels of TNF, IL-1β, IL-12, and IFNγ. We observed that there was a significantly higher level of IL-12 and IFNγ at 7 d*.p.i.* upon *i.p.* infection and nominally, but non-significantly, higher expression of TNF and IL-1β (**Figure 1C**). As the course of infection progressed, the difference in cytokine between the infection routes disappeared. This further supports that the parasite induces inflammation more quickly upon *i.p.* infection. Given the normalization of the immune response starting around 14 d*.p.i.* implies that once the adaptive immune response is initiated, the systemic cytokine production reaches a level of saturation. As *T. gondii* infection progresses, most of the parasites are cleared from the periphery, persisting in muscle cells or within neurons in the CNS (44). To determine if the parasite burden is also associated with the progression of *T. gondii* infection-induced neuroinflammation, we assessed the whole brain cytokine gene expression at 7, 14, and 28 d*.p.i.* in the brain. At 7 and 14 d*.p.i.*, there was no distinguishable difference between cytokine gene expressions (**Figure 1D**). However, as the chronic phase developed at 28 d*.p.i.* we observed a trend toward a higher TNF expression and a significant increase in IL-1β, IFNγ and iNOS expression (**Figure 1D**).

### Long-Term Alterations in the Gut Microbiota Upon *T. gondii* Infection

**Figure 2:**
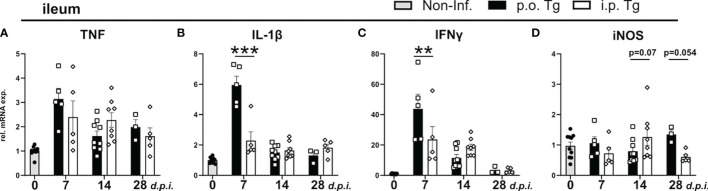
Cytokine levels in the ileum in p.o. vs. i.p. infected mice on day 28 post-infection. Wild-type mice were infected orally (*p.o.* Tg) or *intraperitoneally* (*i.p.* Tg) with *T. gondii* (two cyst, ME49) and **(A-D)** relative mRNA levels of indicated genes were determined in the ileum. Data are normalized to the mean expression of the non-infected group and presented as mean ± SEM. A two-way ANOVA following Fisher’s LSD test was used for statistical analysis (**p<0.01, ***p < 0.001).

Following oral *T. gondii* infection with a high dose of parasites, mice develop acute necrotizing inflammation in the terminal ileum characterized by strong secretion of pro-inflammatory cytokines IL-1β, TNF, and IFNγ (45, 46). However, around d9-10 *p.i.*, intestinal inflammation begins to subside and parasite burden declines in peripheral organs (47), which aligns with our data. To determine how gut homeostasis is altered through the course of infection, we used qRT-PCR to determine the expression of parasite-related immune markers from terminal ileum of infected animals. When analyzing pro-inflammatory cytokine gene expression, we observed a significant increase in gene expression at d7 *p.i.* of IL-1β and IFNγ with no change of TNF or iNOS expression in *p.o.* infected mice when compared to *i.p.* infection (**Figures 2A–D**). This aligns with the observed difference of parasite burden at d7 *p.i.* Of note, there was a trend for a reduced iNOS expression at d14 and increased expression at d28 *p.i.* in *p.o.* infected mice compared to *i.p.* (**Figure 2D**). iNOS is able to control aberrant microbiota growth (48), and the trend of increased expression of iNOS may be a sign of disrupted microbiota in *p.o.* infected mice.

**Figure 3:**
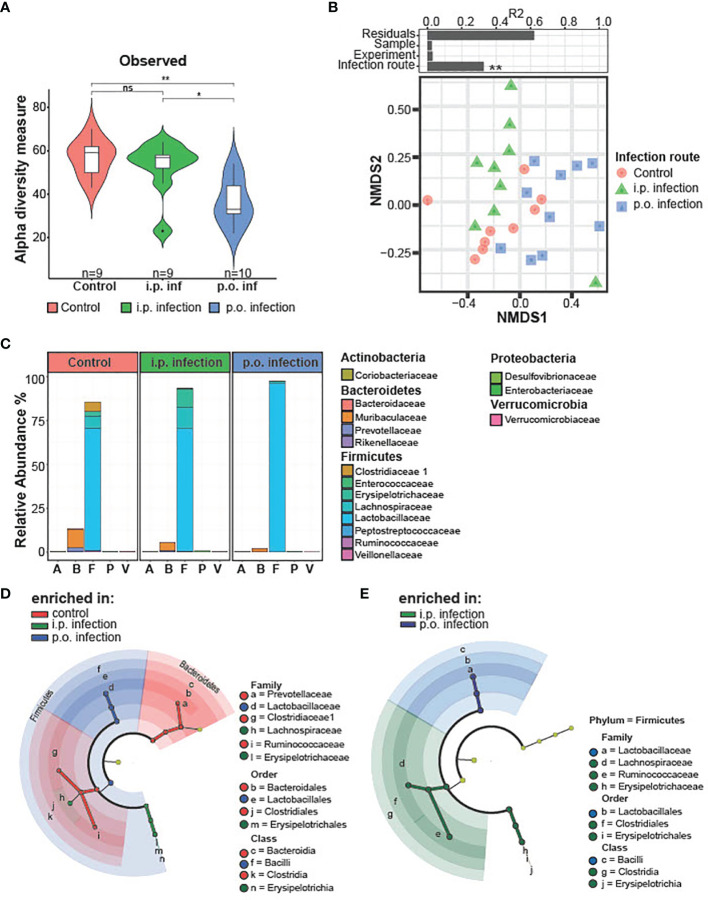
Microbiota composition on day 28 post-infection *i.p.* vs. *p.o.* challenge. Small intestinal microbiota was analyzed using 16S rRNA gene sequencing after 28 days of oral and *intraperitoneal T. gondii* infection. Analyzed samples (n = 9/group and time point) had a minimum sequencing depth of 1000 reads and a mean sequencing depth of 24,946.4 reads. **(A)** Alpha diversity in control and orally infected mice was determined using Chao1 and Shannon index. P-values indicated represent a Mann-Whitney U test comparison between groups with ns, non-significant, **p<0.01. **(B)** Non-metric multidimensional scaling (NMDS) ordination analysis of small intestinal microbiota composition was performed using Bray–Curtis distances. Individual effect size of tested covariates is indicated. To calculate the variance explained by individual factors such as sample, experiment or group a permutational multivariate analysis of variance (ADONIS) was used. *p < 0.05. A significant effect was dedicated when p < 0.05 and R2 > 0.10 (equivalent to 10% of explained variance). **(C)** Relative abundance of the average microbiome composition was determined at the family level. Phylum and families are indicated. **(D)** Statistically significant differences on family levels in small intestinal microbiota composition between control and orally infected mice or **(E)** orally and *i.p.* infected mice. Data were analyzed using linear discriminant analysis (LDA) effect size (LEfSe) method (Kruskal–Wallis test with p < 0.05 and LDA scores > 3.0).

The effect of *T. gondii* infection on the populations of the ileal microbiome during the early phase of infection (d0-d8 *p.i.*) have been well described (30, 49–51). However, given that early *T. gondii* infection is associated with an acute inflammation in the ileum that disrupts the resident microbial communities, we investigated if this early gut disturbance leads to long lasting alterations of the ileal gut microbiota (49, 51). Therefore, we isolated the ileum contents of mice d28 *p.i.* and analyzed them *via* 16S rRNA amplicon sequencing. When assessing the alpha diversity, we found that *p.o.*, and not *i.p.*, *T. gondii* infected mice had a significant reduction in species diversity (**Figure 3A**). Non-metric multidimensional scaling (NMDS) ordination analysis of ileal microbiota composition revealed that the infection route had a significant effect as *T. gondii* infected mice clustered distinct from controls independent of the infection route (**Figure 3B**). To further assess the microbiota composition, we compared the relative abundances of bacteria at the level of phylum and family (**Figure 3C**). *T. gondii*-infected mice displayed a strong reduction in families of the phylum Bacteroidetes and a substantial increase in the Firmicutes phylum, greatly skewing the Bacteroidetes/Firmicutes ratio. Of note, a loss of several Firmicutes families was also observed in infected microbiomes that were replaced by members of the *Lactobacillaceae* family upon *p.o.* infection, while *i.p.* infection resulted in the expansion of the *Erysipelotrichaceae* and *Lachnospiraceae* families. Using the linear discriminant analysis (LDA) effect size (LEfSe) method, microbiome changes were further evaluated (**Figures 3D, E**; **Supplementary Figure 1**). In agreement to previous studies, we observed increases in the gram-negative, proteobacteria family *Enterobacteriaceae* (49, 51). In particular, we detected an increase of the genus *Escherichia*/*Shigella* (**Supplementary Figures 1A–C**), which is known to contain commensal species that aggravate intestinal inflammation (51). There were *i.p.* infection-specific changes to the microbiome with an increased abundance of the Proteobacteria *Desulfovibrio* and Firmicutes *Turicibacter*. Further family-level comparison of naïve and *T. gondii*-infected mice revealed a reduced abundance of many commensal species upon infection (**Supplementary Figures 1D, E**). Of note, there was a loss in the abundance of known beneficial, gram-positive families *Clostridiaceae, Lachnospiraceae, Erysipelotrichaceae* and *Ruminococceae*; as well as gram-negative *Bacteriodetes* families *Muribaculaceae andPrevotellaceae* (**Supplementary Figures 1D, E**). These changes highlight that *T. gondii* infection leads to a disruption of the microbial composition that persists into the chronic stage of infection. This microbial imbalance is highlighted by increases in the abundance of potentially proinflammatory proteobacteria species and a reduction of beneficial commensal species. If and how this microbial imbalance affects the later stages of infection and neuroinflammation remains to be investigated.

### Peripheral Immune Response in *p.o.* and *i.p.* Infected Mice Over the Course of Toxoplasmosis

**Figure 4:**
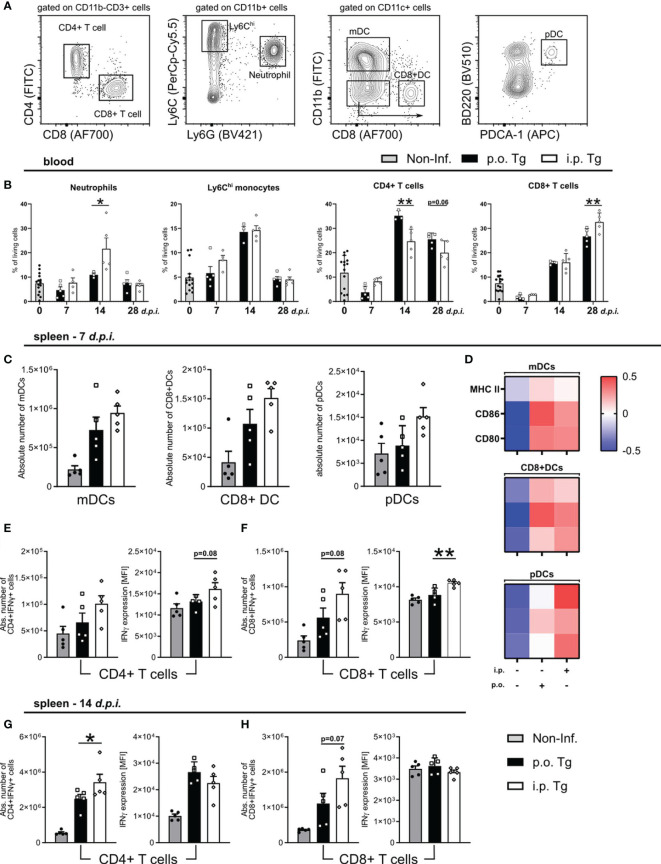
Early T cell response in spleens of mice at day 7 and 14 post-infection. Wild-type mice were infected orally (*p.o.* Tg) or *intraperitoneally* (*i.p.* Tg) with *T. gondii* (two cyst, ME49). Immune cells were isolated from the blood and spleens of naïve (Non-Inf.) and *T. gondii* infected animals and analyzed by flow cytometry. **(A)** Representative gating strategy for myeloid and lymphoid populations (orally infected, 14 d*.p.i.* spleen shown). Viable single cells were chosen for further characterization of CD11b^+^Ly6G^+^ Neutrophils, CD11b^+^Ly6G^-^Ly6C^hi^ monocytes, CD11b^-^CD3^+^CD4^+^ and CD11b^-^CD3^+^CD8^+^ T cells. CD11c^+^ cells were further separated into monocyte-derived DCs (mDC), CD8^+^ DCs and plasmacytoid DCs (pDC). Frequency or absolute number of indicated cell types in the **(B)** blood and **(C)** spleen. **(D)** Heatmaps show relative surface expression of MHC II, CD86, and CD80 in spleen-derived cells at day 7 *p.i.* Quantification of IFNγ production by splenic CD4^+^ and CD8^+^ T cells in infected mice at **(E, F)** 7 and **(G, H)** 14 d*.p.i.* IFNγ expression levels were quantified by the MFI expression of the respective fluorochrome. Data are presented as means + SEM. Differences between groups were analyzed by one-way ANOVA followed by Tukey’s multiple comparisons test (*p<0.05, **p<0.01).

To characterize the immune response based on the infection route, we assessed how the frequency of circulating neutrophils, Ly6C^hi^ monocytes, CD4^+^ and CD8^+^ T, immune cell populations crucial for parasite control, changed over the course of infection using flow cytometry (**Figure 4A**). Consistent with the serum cytokine levels, there was a significant increase of the Ly6G^+^ neutrophil populations on d14 *p.i.* in the blood of *i.p.* infected mice compared to *p.o.* (**Figure 4B**). There were no differences to Ly6C^hi^ inflammatory monocytes in circulation as the infection progressed (**Figure 4B**). There was a skewing of the CD4/CD8 T cell response between the different infection routes. Orally infected mice displayed an increased CD4^+^ T cell population significantly at d14 *p.i.* and with a trend at d28 *p.i.* whereas *i.p.* infected mice exhibited a higher CD8^+^ T cell population at d28 *p.i.* (**Figure 4B**). These results support a more severe course of Toxoplasmosis upon *i.p.* infection.

Since we observed a skewing of the T cell response, and a stronger Th1 associated immune response upon *i.p.* infection, we next investigated if there was a difference in the induction and activation of T cells. At 7 d*.p.i.*, we observed comparable parasite numbers in the spleens of both infection routes, so we decided to assess splenic DC maturation as well as IFNγ production by CD4 and CD8 T cells *via* flow cytometry. We characterized three DC subpopulations. After gating for CD11c^+^ cells, we identified: CD11b^+^CD8^-^ monocyte-derived DCs (mDCs), CD11b^-^CD8^+^ DCs (CD8^+^ DCs), and CD11b^-^CD8^-^B220^+^PDCA-1^+^ plasmacytoid DCs (pDCs) (**Figure 4A**). We observed a non-significant increase in the total number of each DC subpopulation in *i.p.* infected spleen (**Figure 4C**). To assess DC maturation, we measured the surface expression of MHC II, CD86, and CD80. The expression of MHC II, CD86, and CD80 was not differentiable between infection routes for mDCs and CD8^+^ DCs, whereas there was a higher expression on pDCs in *i.p.* infected mice (**Figure 4D**). pDCs, known to release high levels of Type-I interferons, are likely contributing to the exacerbation of disease course in *i.p.* mice. To determine the IFNγ T cell response, T cells were isolated from the spleens of mice and stimulated with *toxoplasma* lysate antigen (TLA), and their IFNγ production was measured *via* intracellular flow cytometric analysis. On d7 *p.i.*, we detected a trend for increased CD8^+^IFNγ^+^ T cells in *i.p.* spleens with no detectable difference in the number CD4^+^IFNγ^+^ T cells (**Figures 4E, F**). When detecting the amount of IFNγ produced (via MFI expression), we found a trend for higher IFNγ production in CD4^+^IFNγ^+^ T cells and a significant increase in CD8^+^IFNγ^+^ T cells (**Figures 4E, F**). By d14 *p.i.*, there continued to be significantly more CD4^+^IFNγ^+^ and a trend for more CD8^+^IFNγ^+^ T cells. However, when assessing the amount of IFNγ produced, there were no detectable differences for either cell type when compared between infection routes (**Figures 4G, H**). These results suggest that there is an earlier activation of effector T cells in *i.p.* infected animals, which indicates a faster parasite spread upon *i.p.* injection.

### Enhanced IFNγ Production by T Cells in *i.p.* Infected Mice

**Figure 5:**
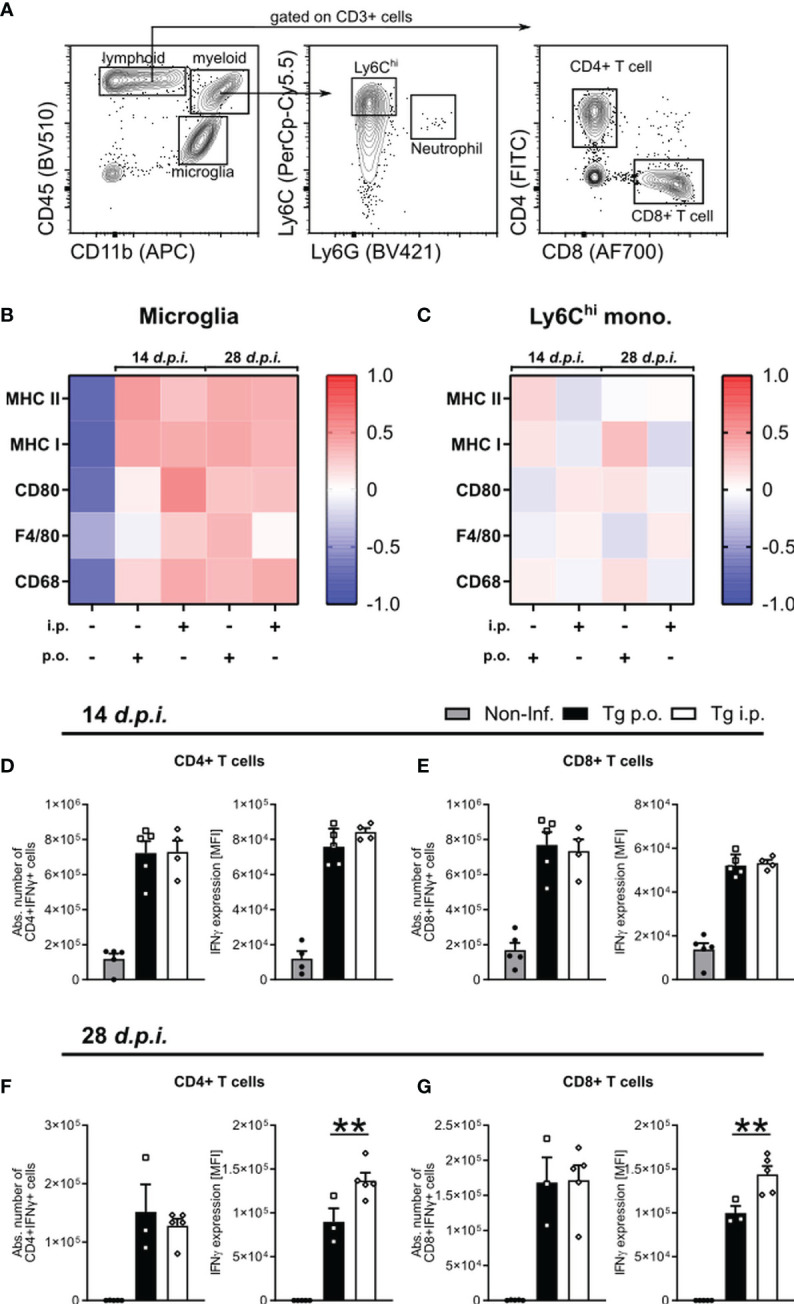
The development of neuroinflammation in *p.o.* vs. *i.p.* infected mice. Immune cells were isolated from the brains of naïve (Non-Inf.) mice and mice after 14 and 28 d*.p.i.* **(A)** Representative gating strategy for resident and recruited immune cell populations in the brain (orally infected, 14 d*.p.i.* brain shown). Following viability staining and the basic FSC/SSC gating, viable single cells were separated by their expression CD45 and CD11b. CD11b^+^CD45^int^ microglia, CD11b^+^CD45^hi^Ly6G^-^Ly6C^hi^ inflammatory monocytes, CD11b^-^CD45^+^CD3^+^ -CD4^+^ and -CD8^+^ T cells were identified. **(B, C)** Heat map plots of the relative surface expression of MHC II, MHC I, CD80, F4/80, and CD68 in naïve, *p.o.* and *i.p.* infected mice at days 14 and 28 *p.i.* For **(B)** microglia and **(C)** Ly6C^hi^ inflammatory monocytes, the median fluorescence intensities (MFI) of each marker were normalized to the overall mean for each respective marker. The quantification of splenic IFNγ production by CD4^+^ and CD8^+^ T cells in mice infected at **(D, E)** 7 and **(F, G)** 14 d*.p.i.* The absolute number of IFNγ^+^ T cells was calculated as a portion of the total living cells. IFNγ expression levels were quantified by the MFI expression of the respective fluorochrome. Positive expression was determined by a matched isotype control. Symbols represent individual animals; columns represent means and error bars represent + SEM. A one-way ANOVA followed by Tukey’s correction for multiple comparisons (**p<0.01).

*T. gondii* infection-induced neuroinflammation is characterized by an early activation of the resident glia and subsequent recruitment of circulating monocytes and effector T cells (49, 51).To determine whether the route of infection affects the development of the neuroinflammatory reponse, we compared the activation status of microglia and recruited Ly6C^hi^ inflammatory monocytes as well as the IFNγ production by CD4 and CD8 T cells (**Figure 5A**). Microglia and monocyte activation was assessed *via* the surface expression on days 14 or 28 p.i. of MHC II, MHC I, CD80, F4/80 and CD68. For both microglia and ly6C^hi^ monocytes, we did not observe any significant differences between *p.o.* or *i.p.* infected mice on days 7 or 14 *p.i. via* Flow cytometric analysis of signature activation marker*s* (**Figures 5B, C**). To control of parasite replication and reactivation of toxoplasmosis, the combined role of effector CD4 and CD8 T cells is required. When looking at the initial recruitment and effector function of both T cells at d14 *p.i.*, we observed no difference in the total number of recruited CD4^+^IFNγ^+^ and CD8^+^IFNγ^+^ T cells or their production of IFNγ (**Figures 5D, E**). As the infection progresses to d28 *p.i.*, there is comparable total numbers of IFNγ^+^ CD4 and CD8 T cells between infection routes, whereas both CD4^+^IFNγ^+^ and CD8^+^IFNγ^+^ cells are producing significantly higher amounts of IFNγ upon *i.p.* infection compared to *p.o.* (**Figures 5F, G**). An increased IFNγ production aligns with the worsened disease course observed in *i.p.* infected animals. Whether this increased severity is due to an increased parasite number or a less effective immune control of the parasite is still to be determined. Together, these results highlight a stronger disease course of Toxoplasmosis with increased numbers of neutrophils, pDC activity, and effector T cell IFNγ production, all known to strongly influence the severity of diseases (52, 53).

### Persisting Decrease in Cortical Synaptic Gene Expression in *i.p.* Infected Mice

**Figure 6:**
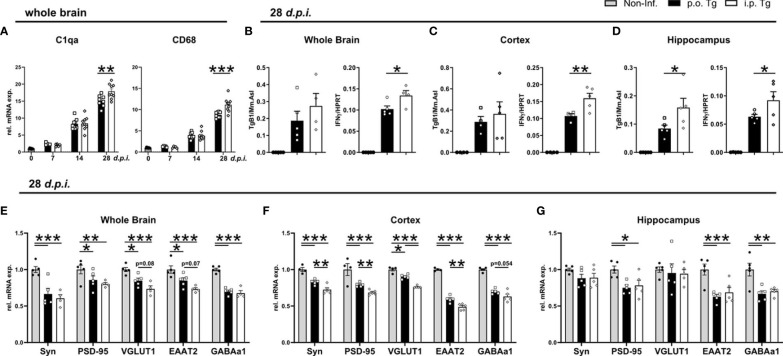
Increased IFNγ gene expression and reduction of synapse-associated gene expression in the cortex of *i.p.* infected mice. Mice were infected *p.o.* and *i.p.* with two cysts of T. gondii for 28 days. **(A)** Relative mRNA levels of synaptic pruning associated genes, C1qa and CD68. **(B–D)** Parasite burden (TgB1 expression) and IFNγ levels in the whole brain, cortex, and hippocampus of *p.o.* and *i.p.* infected animals. Expression of TgB1 gene normalized to the reference gene Mm. Asl. mRNA levels were normalized to the reference gene HPRT. Gene expression in naïve (Non-Inf.), *p.o.* (*p.o.* Tg) and *i.p.* infected (*i.p.* Tg) mice in the early chronic phase (28 d.p.i.) shown for synaptic genes in **(E)** whole brain, **(F)** cortex, and **(G)** hippocampus. Relative gene expression assessed by RT-qPCR analysis. Expression of target genes was normalized to the expression of the reference gene HPRT and subsequently normalized to the mean of naïve mice. Symbols represent individual animals; columns represent means and error bars represent + SEM. A two-way ANOVA followed by Tukey’s correction for multiple comparisons (*p<0.05, **p<0.01, ***p<0.001).

We have previously demonstrated that *T. gondii*-induced neuroinflammation and its inflammatory milieu affect the synaptic protein composition (28, 29), and particularly glutamatergic neurotransmission (54) through chronic infection, we next set out to investigate if the altered inflammatory milieu, especially the enhanced IFNγ production, influenced the gene expression of synaptic markers between infected mice. We previously described and IFNγ-dependent alteration of synaptic composition, regardless of large increases in parasite burden (28). One distinct mechanism for alterations of synaptic compositions is synaptic pruning (55). To determine if there is an alteration in synaptic pruning activity between infection routes, we assessed the gene expression of key synaptic pruning markers C1qa and CD68, markers linked with ‘eat-me signals’ (56–58). When measuring gene expression at days 7, 14, and 28 *p.i.*, we observed that there was no difference in the gene expression at d7 and 14 *p.i.* whereas there was significantly higher expression of both C1qa and CD68 in *i.p.* infected mice at d28 *p.i.* (**Figure 6A**). To determine if there is a brain region-specific difference between infection routes, we investigated the parasite burden and IFNγ expression in the whole brain, cortex, and hippocampus of mice at d28 *p.i.* Assessing parasite burden, we observed a non-significant increase in the parasite burden of whole brain, no change in the cortex, and a significantly higher parasite burden in the hippocampus of i.p. vs. p.o. infected mice (**Figures 6B–D**). IFNγ gene expression, was significantly higher in whole brain as well as in both cortex and hippocampus (**Figures 6B–D**). To assess changes in synaptic composition, we assessed expression of the pre-synaptic vesicle protein synaptophysin (Syp), the post-synaptic scaffolding protein PSD-95 encoded by the *Dlg4* gene, the glutamate transporter EAAT2 encoded by the *Slc1a2* gene, the vesicular glutamate transporter 1 (VGLUT1), and the α1 subunit of the GABA-A receptor (GABAAα1) encoded by the *Gabra1*. In whole brain, there was a significant reduction of all synapse-associated genes in both *p.o.* and *i.p.* infected mice compared to healthy controls and a trend toward a stronger reduction of the expression of VGLUT1 and EAAT2 upon *i.p.* compared to *p.o.* infection (**Figure 6E**). These results align with previous studies showing a similar influence of infection of synaptic gene expression (28, 29). The cortex similarly displayed a substantial reduction in gene expression for all markers upon infection with either *p.o.* or *i.p.* injections. However, when comparing *p.o.* vs. *i.p.* infection, there was a stronger reduction of gene expression for all genes upon *i.p.* infection (**Figure 6F**). In contrast, in the hippocampus, there were no detectable differences upon p.o. or i.p. infection in gene expression of Syn and VGLUT1 (**Figure 6G**). The gene expression of PSD-95, EAAT2, and GABAa1 was significantly reduced upon both *p.o.* and *i.p.* infection compared to uninfected controls, with no changes between the infection routes (**Figure 6G**). These results highlight that there is a more severe influence on the synaptic composition of the cortex upon *i.p.* infection, which aligns with the enhanced disease course observed in these animals.

## Discussion

When a pathogen is ingested, it is immediately subjected to a plethora of defenses: high acidity in the stomach, digestive enzymes in the duodenum, resident microbiota, as well as immune cells to name just a few. However, despite all these defenses, many pathogens are adapted to this environment and have developed mechanisms to survive (59–61). Although the natural way of infection with the common parasite *T. gondii* is the oral ingestion, experimental models apply both *p.o.* and *i.p.* challenges. The unique cell composition in the peritoneal cavity is responsible for a distinct immune response toward pathogens, which strongly differs from ileal immune mechanisms (62). Our data presented here highlight a more severe course of disease progression in *i.p.* infected mice, characterized by an increased weight loss, faster parasite spread, and a more pronounced inflammatory response starting in the acute throughout to the chronic stage of infection. This is associated with an increased loss of gene expression for key synaptic markers involved in glutamatergic signaling throughout the brain, especially in cortical tissue.

Previous studies reported that *T. gondii* infection often leads to weight loss due to anorexia and dehydration (63) as well as chronic cachexia (64). In our experiments, orally infected mice displayed an earlier weight loss but with a better recovery following resolution of the acute response compared to *i.p.* infected mice. Possibly due to differences in the barrier of entry between infection routes, or a faster exposure to migratory cells/circulation in *i.p.* infection, there is a more severe disease course upon *i.p.* infection. Upon oral infection, the parasites cross at the intestinal epithelium, where there could be direct competition from the local microbiome, the very acidic pH of the stomach and duodenum, the fluid dynamics involved in digestive propulsion, and the distinct immune response in the gut-assiciated lymphoid tissue. There is a significantly higher impedance to that initial spread, and an increased time delay for when parasites cross into the body itself. This is evidenced by observing a majority of the detected parasite burden being present in *p.o.* infected ileal tissue on d7 *p.i.*

Invasion of the intestinal epithelium leads to intense inflammation and a dramatic disruption of the normal microbial ecosystem that resides in the healthy gut. One characteristic change in the microbiome during infection is the overgrowth of *Escherichia coli* (*E. coli*) or related bacteria and loss of control of commensals which leads to the spread of gut bacteria to peripheral organs and subsequent sepsis (48). We observed an increase in the abundance of proteobacteria populations after *T. gondii* infection. The growth of proteobacteria such as *E. coli* and other proteobacteria species has been shown to aggravate inflammation in the gut as well as peripheral tissues by targeting intestinal epithelial cells in parasite infection, pancreatitis, and models of irritable bowel disease (45, 51, 65–67). Our results align with increased abundance of proteobacteria populations, especially that of the genus *Escherichia-Shigella*. However, the observed increase in abundance upon low-dose infection was much less pronounced compared to high-dose *T. gondii* infection studies (49, 51, 68). Hand et al., described that during a gastrointestinal infection, tolerance to commensals is lost and microbiota-specific T cells are activated and differentiate to inflammatory effector cells (47). Furthermore, these T cells are comparable in function and phenotype to pathogen-specific T cells. This is a likely explanation for the observed bias toward a higher CD4^+^ T cell response in *p.o.* infected mice. Further experiments are needed to determine if there is a population of non-*T. gondii* specific T cells persisting in *i.p.* infected mice. We detected infection route-specific changes, such as the growth of the delta-proteobacteria family Desulfovibrio. The difference in microbial change is likely due to a difference in the ongoing immune response once inflammation occurs in the intestine. In *i.p.* infection, this appears to be well after inflammation has begun to develop toward its Th1 response. Moreover, we previously described comparable results upon co-infection of *T. gondii* with the intestinal nematode *H. polygryus* (*H. polygyrus*), a strong Th2-inducing pathogen that is associated with immunosuppression (69). In co-infected mice, there is an activation Th1 CD4^+^ T cells that specifically respond to H. polygyrus antigen with a corresponding inability to generate Th2 CD4^+^ T cells. Similar to our results here, these co-infected mice with non-*T.gondii*-specific CD4 Th1 T cells correlated with an exacerbated disease outcome highlighted by worsened weight loss, an enhanced IFNγ T cell immune response in the brain, and associated brain pathology (28). Thus, understanding the mechanisms involved in regulating pathogen infection and the adjuvant effect of secondary or peripheral infections are of great importance.

Once parasites penetrate the BBB and enter the brain, they invade resident brain cells and transform into cysts. The tachyzoite-to-bradyzoite conversion, key for beginning the chronic phase of infection, is a stress-mediated response that corresponds to a slowing of growth of the parasite (70, 71). The onset of adaptive immunity and a parasite specific response is crucial to trigger the conversion to the bradyzoite form. Adaptive immunity relies heavily on antigen-presenting cells (APCs), through their activation of T cells in secondary lymphoid organs. When we assessed DC and effector T cell populations in the spleen, we found no difference in APC function of DCs, however there was increased activity of pDCs in *i.p.* infected mice. pDCs, known to release high levels of Type I interferons, are potentially contributing to the worsening disease course observed (72). Furthermore, we detected that T cells isolated from spleens on d7 and d14 *p.i.* increased IFNγ expression in CD4^+^ and CD8^+^ T cells of *i.p.* infected mice on d7 *p.i.* suggesting an earlier polarization to effector T cells. Further, by d14 *p.i.* there was no difference in IFNγ expression, implying that once T cells are activated, they do not differ in their Th1 function depending on infection route. The total number of both CD4^+^ and CD8^+^ effector T cells in the spleens at d14 *p.i* was increased. This further supports that in *i.p.* infected mice, the parasite begins disseminating faster, encounters the immune system earlier, and is quicker to initiate adaptive immunity. With an earlier adaptive Th1 immune response, one would expect better clearance of the parasite. However, we see enhanced pro-inflammatory cytokine expression in the brain as well as enhanced effector T cell activity *via* increased IFNγ expression. Thus, it is likely that the worsened course of toxoplasmosis in *i.p.* infected mice may be due to the increased spread during the initial injection of parasite. The immune system is being challenged in a greater area of effect and likely not as efficient in parasite clearance. This is partially supported by the observed differences in parasite burden, however, consequently, more organs should be investigated. Recently, in different model systems, comparable inflammatory pattern was reported (73, 74). *L. interrogans* infection induces a potent acute myeloid response that requires a robust adaptive T cell response to control its spread, but ultimately inflammation is not resolved and infection persists indefinitely.

*T. gondii* has an uncanny ability to persist in the CNS, being responsible for basal neuroinflammation. The *T. gondii*–CNS interaction associated with rodent behavioral changes, increased risk for schizophrenia in infected humans, and profound alterations glutamatergic neurotransmission and associated excitatory/inhibitory neural signaling (12, 29, 54, 75, 76). These changes have partially been reversed through Sulfadiazine and Pyrimethamine therapy (77). It has been shown that IFNγ mediates much of these associated CNS changes after infection, such as loss of GABA transporter localization, reduced expression of key synaptic components, and displacement of synapses by microglia (76). In agreement with these findings, we identified a loss of gene expression in key synaptic proteins upon *T. gondii* infection, regardless of the infection route. One proposed mechanism for synaptic loss is enhanced synaptic elimination by microglia. When assessing signature synaptic pruning markers, C1qa and CD68, we detected an increase in gene expression persistent through the course of infection. At d28 *p.i.*, this expression was significantly higher in *i.p.* infected mice. However, we did not observe obvious differences in the activation status of microglia or recruited monocyte function between the infection routes. Future studies are necessary to find the cells directly involved in these synaptic changes. Of note, we did observe a trend of stronger loss of expression of VGLUT1 and EAAT2, proteins crucial to synaptic, upon *i.p.* infection. This effect was primarily observed in the cortex, whereas no differences between infection routes were found in the hippocampal region. For all cortical synaptic markers measured, we detected a stronger loss of gene expression in *i.p.* infected animals. A potential explanation for the reduced synaptic gene expression in the hippocampus, even upon *p.o.* infection, may be that the hippocampus is a site of neurogenesis, and that younger neurons, are a preferential host for *T. gondii*.

**Figure 7:**
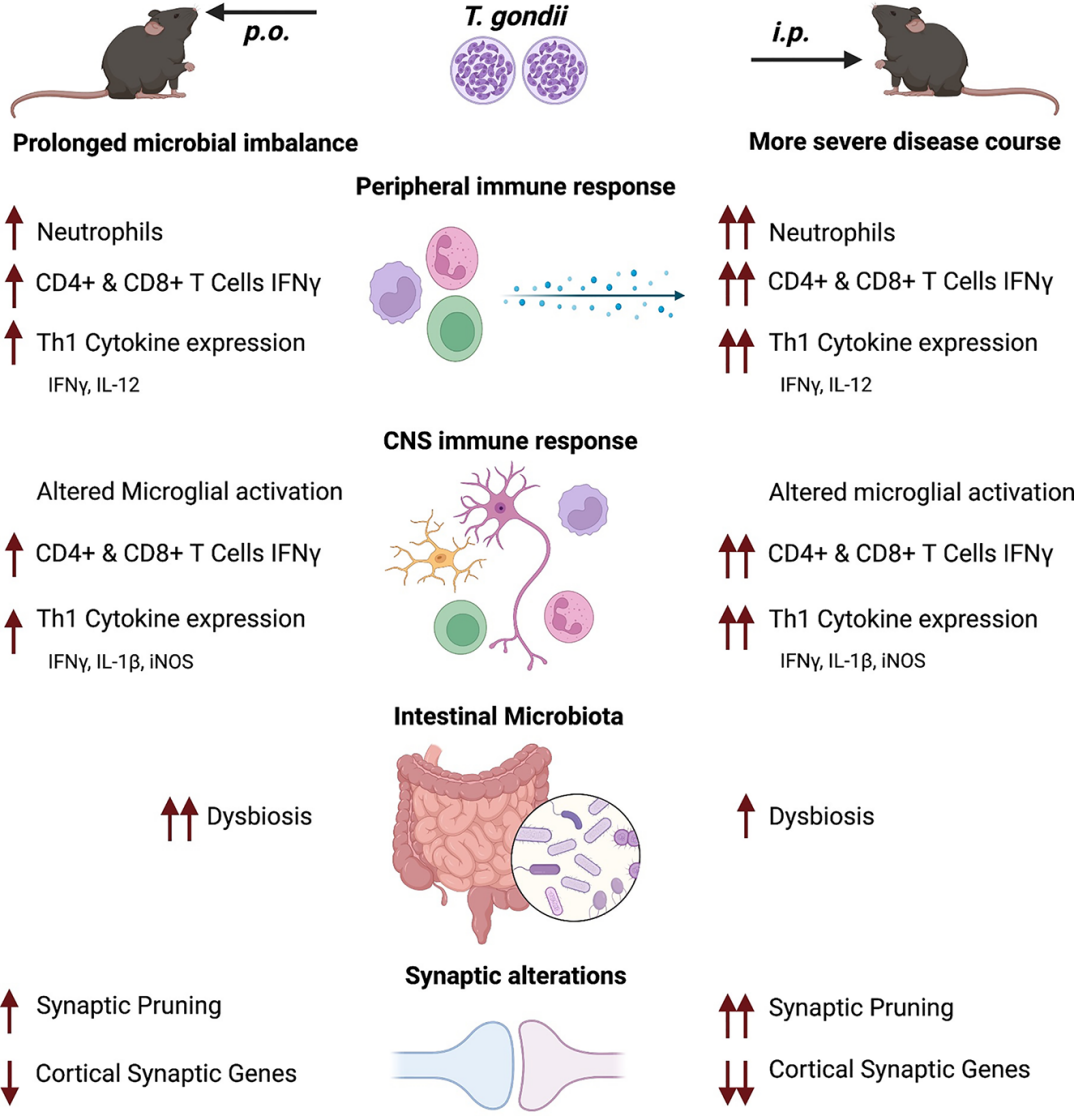
Overview of the *T. gondii* infection disease course after *p.o.* and *i.p.* challenge. Both oral (*p.o.*) or *intraperitoneal* (*i.p.*) infection with the parasite *T. gondii* is followed by a robust peripheral immune response characterized by recruitment of myeloid cells, initiation of the Th1 immune response (IL-12 and IFNγ production) and activation of effector T cells. After dissemination, the parasites reach the brain where they differentiate into cysts, causing persistent chronic neuroinflammation. The observed inflammation is characterized by prolonged microglia activation, enhanced synaptic pruning activity and alterations to neural signaling *via* synaptic alterations. Upon *i.p.* challenge of mice a more severe disease course is established, with an increased weight loss, higher parasite burden and myeloid cell recruitment as well as a stronger IFNγ production by T cells. IFNγ, crucial to parasite control, is produced more by T cells after *i.p.* infection, which is associated with stronger alteration in synaptic marker expression especially in the cortex. *T. gondii* infection disrupts the intestinal microbiome upon infection. *I.p.* infection leads to strong shifts in the microbial composition but is less pronounced than the shifts observed upon *p.o.* infection. This is further exemplified by a loss in microbial diversity of *p.o.* challenged mice that persists well beyond parasite clearance from the intestines.

In this project, we compared the influence of *p.o.* vs. *i.p.* infection route on the course of *T. gondii* infection-induced inflammation and subsequent neuronal homeostasis. Summarized in **Figure 7**, we observed a more severe course of infection in *i.p.* challenged mice that was characterized by an increased weight loss, more rapid parasite spread, and a more pronounced inflammatory response starting in the acute through to chronic infection. The marked inflammatory response resulted in long-term microbial dysbiosis, particularly after *p.o.* challenge. Notably, *i.p.* infection was associated with a distinct persisting loss of gene expression for key synaptic markers involved in glutamatergic signaling in the cortical tissue. We conclude that the route of infection is substantially determining the course of *T. gondii* infection, especially altering neuronal homeostasis.

## Data Availability Statement

The original contributions presented in the study are publicly available. This data can be found here: BioProject, PRJNA821028.

## Ethics Statement

The animal study was reviewed and approved by Landesverwaltungsamt Halle, Sachsen-Anhalt, Germany.

## Author Contributions

TF and AG conducted the experiments. TF analyzed data. LO analyzed microbiome data. TF, JS, TSc, BS, TSt, and IRD interpreted data. TF and IRD wrote the paper. IRD designed and supervised the study. All authors contributed to the article and approved the submitted version.

## Funding

This work was supported by grants from the German Research Foundation to TS (DFG SPP1937, SCHU 2326/2-2), ID (DFG SPP1937, DU1112/5-1 and RTG 2413 SynAGE).

## Conflict of Interest

The authors declare that the research was conducted in the absence of any commercial or financial relationships that could be construed as a potential conflict of interest.

## Publisher’s Note

All claims expressed in this article are solely those of the authors and do not necessarily represent those of their affiliated organizations, or those of the publisher, the editors and the reviewers. Any product that may be evaluated in this article, or claim that may be made by its manufacturer, is not guaranteed or endorsed by the publisher.
