# Supplementary material for: Persisting Microbiota and Neuronal Imbalance Following T. gondii Infection Reliant on the Infection Route
Source: Front Immunol. 2022 Jul 11;13:920658. doi: 10.3389/fimmu.2022.920658 (PMC9311312; doi:10.3389/fimmu.2022.920658)
Supplement: Supplementary file 1 [file DataSheet_1.docx]

Supplementary Material

## Supplementary Figures


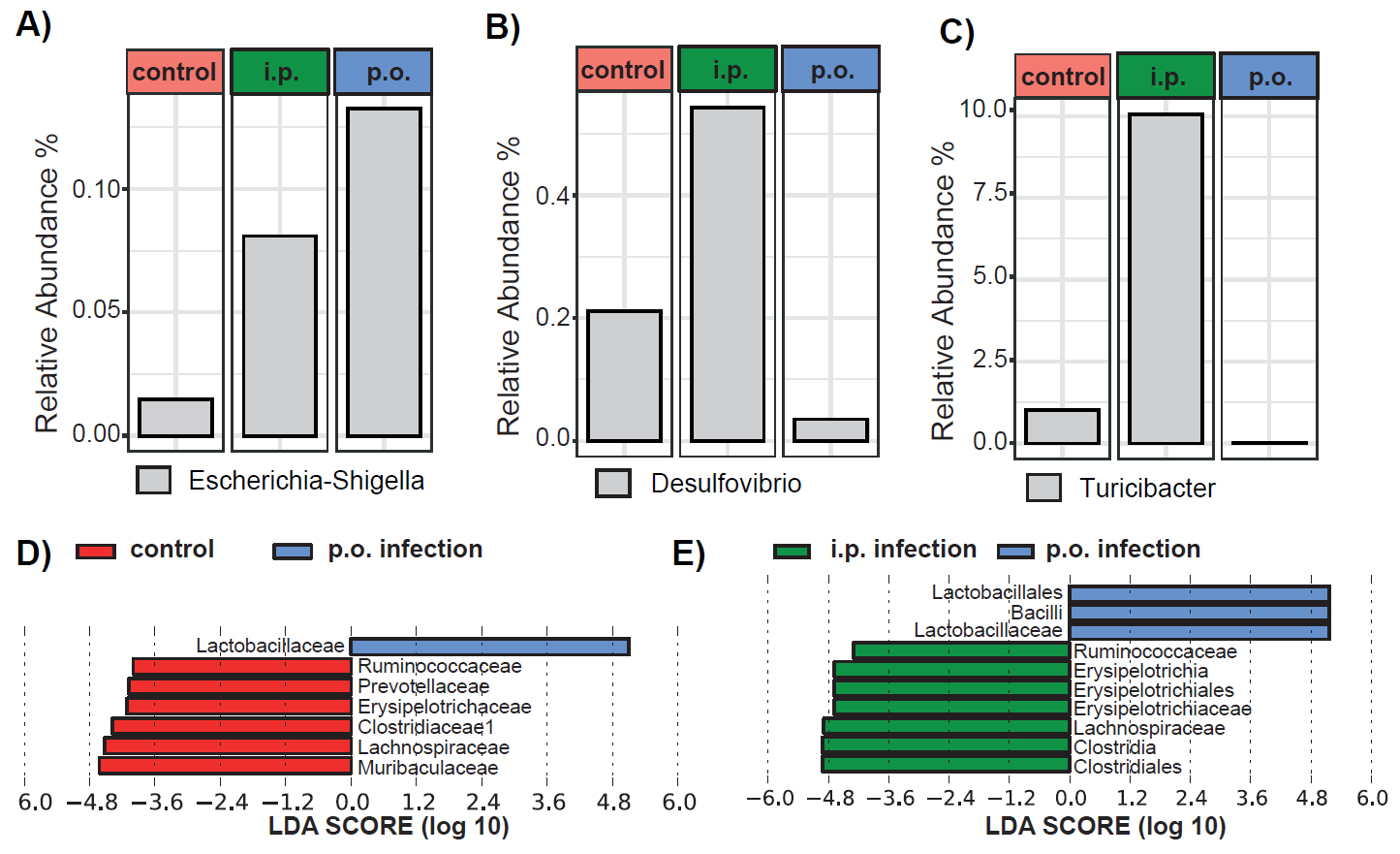


**Supplementary Figure 1: Changes in microbiota composition upon *p.o* and *i.p.* infection**

Small intestinal microbiota was analyzed using 16S rRNA gene sequencing after 28 days of oral or intraperitoneal *T. gondii* infection. Analyzed samples (*n* = 9/group and time point) had a minimum sequencing depth of 1000 reads and a mean sequencing depth of 24,946.4 reads. Relative abundance of the average microbiome composition was determined on family level. Phylum and families are indicated. Statistically significant differences on family levels in small intestinal microbiota composition between control, orally and intraperitoneally infected mice. Data were analyzed using linear discriminant analysis (LDA) effect size (LEfSe) method (Kruskal–Wallis test with p < 0.05 and LDA scores > 3.0).
